# Long-term changes in cardiovascular risk markers during administration of exenatide twice daily or glimepiride: results from the European exenatide study

**DOI:** 10.1186/s12933-015-0279-z

**Published:** 2015-09-04

**Authors:** Rafael Simó, Bruno Guerci, Guntram Schernthaner, Baptist Gallwitz, Juan Rosas-Guzmàn, Francesco Dotta, Andreas Festa, Ming Zhou, Jacek Kiljański

**Affiliations:** CIREDEM, Carlos III Health Institute, Barcelona, Spain; Diabetes Research and Metabolism Unit, Institut de Recerca Hospital Universitari Vall d’Hebron, Barcelona, Spain; Diabetologie, Maladies Metaboliques and Nutrition, Hôpital Brabois, CHU de Nancy, et CIC Inserm, Vandoeuvre Lès Nancy, France; Rudolfstiftung Hospital-Vienna, Vienna, Austria; Medizinische Klinik IV, Universitätsklinikum Tübingen, Tübingen, Germany; Celaya Center for Specialist Medicine, Celaya, Guanajuato Mexico; Diabetes Unit, Policlinico Le Scotte, University of Siena, Siena, Italy; Eli Lilly and Company, Eli Lilly Regional Operations Ges.m.b.H., Vienna, Austria; Bristol-Myers Squibb, Pennington, NJ USA; Eli Lilly, Eli Lilly and Company, Eli Lilly Polska Sp. z o.o., Warsaw, Poland

**Keywords:** Type 2 diabetes, GLP-1 receptor agonist, Exenatide twice daily, Cardiovascular risk, High-sensitivity C-reactive protein

## Abstract

**Objective:**

The risk of cardiovascular morbidity and mortality is significantly increased in patients with diabetes; thus, it is important to determine whether glucose-lowering therapy affects this risk over time. Changes in cardiovascular risk markers were examined in patients with type 2 diabetes treated with exenatide twice daily (a glucagon-like peptide-1 receptor agonist) or glimepiride (a sulfonylurea) added to metformin in the EURopean EXenAtide (EUREXA) study.

**Research design and methods:**

Patients with type 2 diabetes failing metformin were randomized to add-on exenatide twice daily (n = 515) or glimepiride (n = 514) until treatment failure defined by hemoglobin A1C. Anthropomorphic measures, blood pressure (BP), heart rate, lipids, and high-sensitivity C-reactive protein (hsCRP) over time were evaluated.

**Results:**

Over 36 months, twice-daily exenatide was associated with improved body weight (−3.9 kg), waist circumference (−3.6 cm), systolic/diastolic BP (−2.5/−2.6 mmHg), high-density lipoprotein (HDL)-cholesterol (0.05 mmol/L), triglycerides (−0.2 mmol/L), and hsCRP (−1.7 mg/L). Heart rate did not increase (−0.3 beats/minute), and low-density lipoprotein-cholesterol (0.2 mmol/L) and total cholesterol (0.1 mmol/L) increased slightly. Between-group differences were significantly in favor of exenatide for body weight (*P* < 0.0001), waist circumference (*P* < 0.001), systolic BP (*P* < 0.001), diastolic BP (*P* = 0.023), HDL-cholesterol (*P* = 0.001), and hsCRP (*P* = 0.004). Fewer patients randomized to exenatide twice daily versus glimepiride required the addition of at least one antihypertensive (20.4 vs 26.4 %; *P* = 0.026) or lipid-lowering medication (8.4 vs 12.8 %; *P* = 0.025).

**Conclusions:**

Add-on exenatide twice daily was associated with significant, sustained improvement in several cardiovascular risk markers in patients with type 2 diabetes versus glimepiride.

Clinical trial registration: NCT00359762, http://www.ClinicalTrials.gov

## Background

Type 2 diabetes significantly increases the risk of cardiovascular morbidity and mortality [1]. Clinical practice guidelines recommend the use of metformin as first-line therapy for the management of type 2 diabetes [2, 3]. In addition to lowering hemoglobin A1C, long-term data indicate that metformin also may be associated with risk reductions in cardiovascular outcomes [4]. As the majority of patients with type 2 diabetes often fail to achieve or maintain glycemic control with metformin alone, additional second-line therapies are often required [5]. Guideline-recommended add-on therapies to metformin include glucagon-like peptide-1 receptor agonists (GLP-1RAs), sulfonylureas, thiazolidinediones, dipeptidyl peptidase-4 inhibitors, sodium-glucose cotransporter-2 inhibitors, and insulin [2, 3]. Because of a lack of comparative long-term outcomes studies of dual-agent combinations that assess cardiovascular measures, the cardiovascular risk benefits of add-on therapies to metformin are uncertain [6]. The measurement of various markers of cardiovascular risk (e.g., weight, waist circumference, blood pressure [BP], glycemic markers, lipid levels) in long-term studies of safety and efficacy may help to provide some guidance where long-term outcomes data are lacking.

The EURopean EXenAtide (EUREXA) study was the longest randomized, active-controlled, multinational study of a GLP-1RA with comparative treatment for up to 4.5 years with cardiovascular risk marker data collected throughout the trial as secondary endpoints [7]. EUREXA compared the efficacy of exenatide twice daily versus glimepiride as second-line add-on therapy in patients with type 2 diabetes who had inadequate glycemic control with metformin alone. Although patients in randomized controlled trials might differ in several aspects from patients in real-life clinical practice, patients enrolled in EUREXA were similar to typical patients with type 2 diabetes with regard to the prevalence of overweight/obesity, lipid abnormalities, and cardiovascular disease at baseline. Primary data on time to treatment failure for patients in the EUREXA study have been published previously [7]. The objective of the current report was to examine changes in the cardiovascular risk markers, which included waist circumference, BP, serum lipids, and high-sensitivity C-reactive protein (hsCRP, as a surrogate marker of inflammation), over time with exenatide twice daily versus glimepiride.

## Research design and methods

### Study design and patients

EUREXA was a randomized, multicenter, open-label, two-arm parallel, active comparator-controlled trial of adult patients with type 2 diabetes with inadequate glycemic control using metformin [7]. Patients were enrolled in 14 countries (Austria, Czech Republic, Finland, France, Germany, Hungary, Ireland, Israel, Italy, Mexico, Poland, Spain, Switzerland, and the United Kingdom). The study was performed with approval from institutional review boards in line with country-specific regulations, and in compliance with Good Clinical Practice and the principles of the Declaration of Helsinki. All patients provided signed informed consent.

Full inclusion and exclusion criteria were published previously [7]. Briefly, enrolled patients were aged 18–85 years with type 2 diabetes, had a body mass index ≥25 to <40 kg/m^2^ with stable body weight for at least 3 months, were taking stable and maximum tolerated doses of metformin, and had inadequate glycemic control [A1C ≥6.5 % (48 mmol/mol) and <9.0 % (75 mmol/mol)]. Patients with active or untreated malignancy, renal or liver disease or dysfunction, chronic anemia or hemoglobinopathy, proliferative retinopathy or macular edema, or severe gastrointestinal disease were excluded.

Eligible patients were randomized in a 1:1 ratio, with stratification by A1C, to receive exenatide twice daily or glimepiride as add-on to current metformin treatment. Exenatide twice daily was initiated at 5 µg twice daily, increasing to 10 µg twice daily after 4 weeks, and was subcutaneously administered by an injection pen in the abdomen, thigh, or upper arm within 60 min before meals that were at least 6 h apart. The recommended starting dose for glimepiride was 1 mg/day orally immediately before the morning meal, with titration every 4 weeks to the maximum tolerated dose, in accordance with the usual practice of the attending physician. As far as possible, patients continued to use the same formulation and dose of metformin throughout the study. There was no restriction on concomitant therapies, except for other anti-diabetes medications, weight-loss agents, and glucocorticoids.

Patients who experienced inadequate A1C control, defined as A1C concentration >9.0 % (75 mmol/mol) at any time after the first 6 months of randomized treatment or >7.0 % (53 mmol/mol) at two consecutive study visits 3 months apart after the first 6 months of randomized treatment, were discontinued and offered alternative treatment.

### Study assessments

Patients visited the clinic at baseline, Week 4, and at 3-month intervals after baseline until reaching the primary endpoint or study conclusion. A1C was measured every 3 months after baseline. Markers of cardiovascular risk included body weight (measured at baseline, Week 4, and every 3 months after baseline); waist circumference, BP, and heart rate (measured at baseline, Week 4, and every 6 months after baseline); and serum lipids (total cholesterol, low-density lipoprotein (LDL)-cholesterol, high-density lipoprotein (HDL)-cholesterol, and triglycerides) and hsCRP (measured at baseline and 1-year intervals after baseline). Safety assessments included data on hypoglycemic episodes (classified according to the American Diabetes Association recommendations [8]) and concomitant medications collected at each study visit as part of routine collection in the trial.

BP was determined three times, seated and after a 5-min rest, and the mean of the second and third measurements was recorded. All serum lipid and hsCRP assays were carried out at a central laboratory (Interlab GmbH, Munich, Germany). A1C was determined by automated high-performance liquid chromatography (Tosoh Bioscience Inc., San Francisco, CA, USA). Serum concentrations of total cholesterol, HDL-cholesterol, and triglycerides were determined using automated enzymatic colorimetric methods (Roche Diagnostics GmbH, Mannheim, Germany), with LDL-cholesterol calculated using the Friedewald formula, and hsCRP measured by a high-sensitivity particle-enhanced turbidimetric assay (Roche Diagnostics GmbH).

### Statistical analysis

The intent-to-treat (ITT) efficacy population included randomized patients who received at least one dose of study treatment and had baseline and at least one post-baseline measurement of A1C. Analyses of cardiovascular risk markers and safety measurements used an ITT safety population comprising all patients who received at least one dose of study drug. Continuous variables were analyzed by mixed model repeated measures (MMRM), with terms for treatment, study visit, and interactions, and including the baseline value as a covariate. Study visits with at least 25 % of randomized patients remaining were included in MMRM analyses, and least-squares (LS) means and 95 % confidence intervals were derived from the model for each time point. Differences in rates of hypoglycemia, as episodes per year, were analyzed between groups using a negative binomial model. Between-group differences in use of concomitant medications were analyzed using Fisher’s exact test.

Ad hoc analyses for differences between treatment groups in proportions of patients meeting therapeutic goals at their final visit in the study for waist circumference, systolic and diastolic BP (SBP and DBP, respectively), and lipid concentrations were examined using a logistic regression model; therapeutic goals were those recommended by the American Diabetes Association [9].

## Results

### Baseline disposition and clinical characteristics

Patient disposition and baseline characteristics have been previously described [7]. Briefly, of 1029 patients randomized in the study, 515 were assigned to receive exenatide twice daily and 514 were assigned to receive glimepiride as an add-on to metformin. Of patients randomized to exenatide twice daily and glimepiride, 511 and 508 were included in the safety population, respectively. A total of 174 patients in the exenatide arm and 128 patients in the glimepiride arm discontinued treatment; the most common reason for discontinuation in each arm was patient decision (70 and 50 patients, respectively) [7]. Baseline characteristics were similar between the treatment groups except for fewer patients taking antihypertensive and lipid-lowering medications in the exenatide twice-daily group (Table 1). At the start of randomized treatment, antihypertensive and lipid-lowering medications were reported for 65.4 and 45.6 %, respectively, of the exenatide twice-daily group, and 70.3 and 53.4 % of the glimepiride group. Overall, antihypertensives were taken by 72.4 % of patients and lipid-lowering agents were taken by 54.4 % of patients during the study.

**Table 1:** Baseline characteristics of patients in the ITT safety population

	Exenatide twice daily (n = 511)	Glimepiride (n = 508)
Age (year), mean (SD)*	56 (10)	57 (9)
Male/female (%)*	56/44	52/48
Body weight (kg), mean (SD)	92.6 (16.6)	90.9 (15.1)
BMI (kg/m^2^), mean (SD)	32.5 (4.2)	32.2 (4.0)
Waist circumference (cm), mean (SD)	108.3 (11.8)	107.6 (11.5)
Systolic blood pressure (mmHg), mean (SD)	132.9 (15.7)	133.6 (15.4)
Diastolic blood pressure (mmHg), mean (SD)	80.5 (9.4)	79.8 (10.1)
Heart rate (beats per minute), mean (SD)	74.0 (9.3)	74.0 (10.1)
A1C (%), mean (SD)*	7.5 (0.7)	7.4 (0.7)
A1C (mmol/mol), mean (SD)*	58 (7.7)	57 (7.7)
Total cholesterol (mmol/L), mean (SD)	4.7 (1.0)	4.7 (1.0)
LDL-cholesterol (mmol/L), mean (SD)	2.5 (0.8)	2.5 (0.8)
HDL-cholesterol (mmol/L), mean (SD)	1.3 (0.3)	1.3 (0.3)
Triglycerides (mmol/L), mean (SD)	1.9 (1.2)	2.0 (1.3)
High sensitivity C-reactive protein (mg/L), mean (SD)	4.8 (7.7)	4.2 (5.1)
Taking antihypertensive drugs (%)	65.4	70.3
Taking lipid-lowering drugs (%)	45.6	53.4

* Age, sex, and A1C were measured for the ITT efficacy population only as per protocol, exenatide (n = 490), glimepiride (n = 487).* BMI* body mass index,* HDL* high-density lipoprotein, *ITT* intent-to-treat,* LDL* low-density lipoprotein, *SD* standard deviation

### Body weight and waist circumference changes over time in exenatide twice daily- and glimepiride-treated patients

**Fig. 1:**
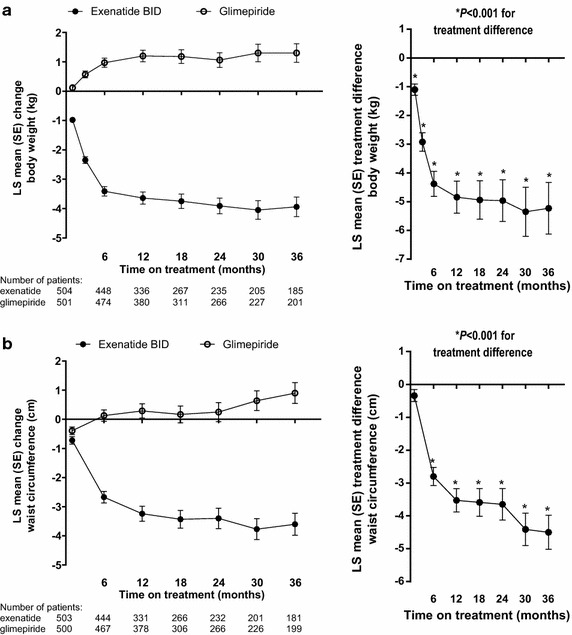
LS mean (SE) change from baseline (*left pane*l) and treatment difference (*right panel*, exenatide twice daily − glimepiride) in body weight (**a**) and waist circumference (**b**) in randomized patients. *BID* twice daily, *LS* least-squares, *SE* standard error

Body weight decreased from baseline in the exenatide twice-daily group and increased in the glimepiride group at 36 months (Table 2; Fig. 1a). The between-group difference in the change from baseline was significantly in favor of exenatide twice daily at each visit from 6 to 36 months (*P* < 0.0001). Analysis by sex showed similar trends between males and females for body weight change and between-group differences. Significant between-group differences in favor of exenatide twice daily were observed in both males [LS mean (standard error [SE]), −5.3 (0.63) kg; *P* < 0.0001] and females [−5.2 (0.68) kg; *P* < 0.0001].

**Table 2:** LS mean (SE) change from baseline in cardiovascular risk markers at 36 months

	Exenatide (n = 511)	Glimepiride (n = 508)	*P* value for treatment difference
Body weight (kg)			
n	185	201	
LS mean (SE)	−3.9 (0.33)	1.3 (0.32)	
95 % CI	−4.6, −3.3	0.7, 1.9	
Treatment difference	−5.2 (0.46)		<0.0001
Waist circumference (cm)			
n	181	199	
LS mean (SE)	−3.6 (0.38)	0.9 (0.36)	
95 % CI	−4.4, −2.9	0.2, 1.6	
Treatment difference	−4.5 (0.28)		<0.001
Systolic blood pressure (mmHg)			
n	183	203	
LS mean (SE)	−2.5 (0.89)	2.8 (0.85)	
95 % CI	−4.2, −0.7	1.1, 4.4	
Treatment difference	−5.2 (1.23)		<0.001
Diastolic blood pressure (mmHg)			
n	183	203	
LS mean (SE)	−2.6 (0.55)	−0.9 (0.52)	
95 % CI	−3.7, –1.5	−1.9, 0.2	
Treatment difference	−1.7 (0.75)		0.023
Heart rate (beats per minute)			
n	181	199	
LS mean (SE)	−0.3 (0.58)	0.4 (0.56)	
95 % CI	−1.4, 0.9	−0.7, 1.5	
Treatment difference	−0.7 (0.81)		0.374
Total cholesterol (mmol/L)			
n	178	193	
LS mean (SE)	0.1 (0.06)	0.1 (0.05)	
95 % CI	0.0, 0.3	0.0, 0.2	
Treatment difference	0.0 (0.08)		0.791
LDL-cholesterol (mmol/L)			
n	171	184	
LS mean (SE)	0.2 (0.05)	0.1 (0.05)	
95 % CI	0.1, 0.3	0.0, 0.2	
Treatment difference	0.1 (0.07)		0.304
HDL-cholesterol (mmol/L)			
n	178	195	
LS mean (SE)	0.05 (0.01)	−0.02 (0.01)	
95 % CI	0.02, 0.07	−0.04, 0.01	
Treatment difference	0.06 (0.02)		0.001
Triglycerides (mmol/L)			
n	178	193	
LS mean (SE)	−0.2 (0.06)	0.1 (0.06)	
95 % CI	−0.3, −0.1	−0.1, 0.2	
Treatment difference	−0.3 (0.09)		0.004

*CI* confidence interval,* HDL* high-density lipoprotein,* LDL* low-density lipoprotein, *LS* least-squares, *SE* standard error

Waist circumference decreased from baseline in the exenatide twice-daily group and increased in the glimepiride group at 36 months (Table 2; Fig. 1b). The between-group difference in the change from baseline was significant at each visit from 6 to 36 months in favor of exenatide twice daily (*P* < 0.001). Between-group differences also were significant in both males (LS mean [SE], –5.0 [0.71] cm; *P* < 0.0001) and females [–4.0 (0.76) cm; *P* < 0.0001] at 36 months.

### BP and heart rate changes over time in exenatide twice daily- and glimepiride-treated patients

**Fig. 2:**
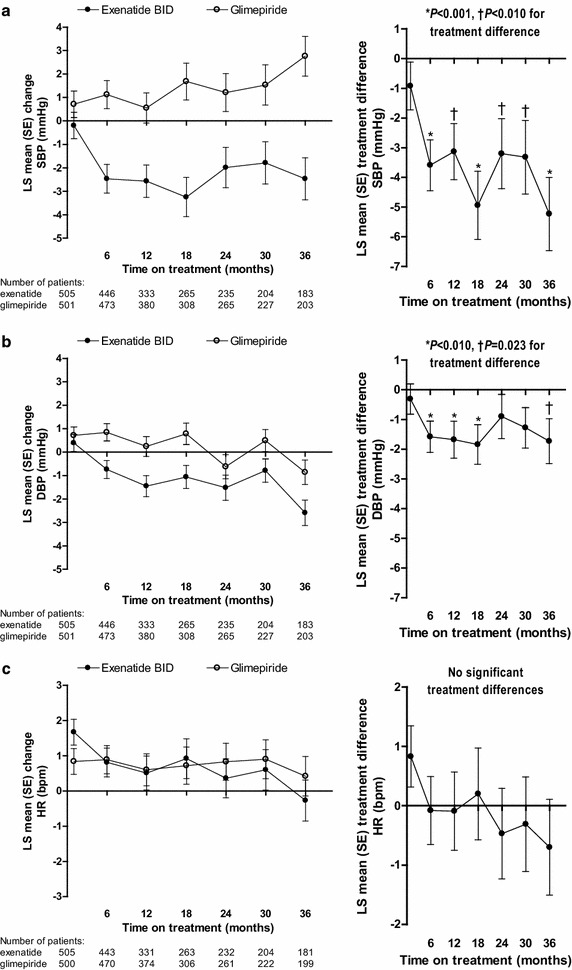
LS mean (SE) change from baseline (*left panel*) and treatment difference (*right panel*, exenatide twice daily − glimepiride) in systolic blood pressure (**a**), diastolic blood pressure (**b**), and heart rate (**c**) in randomized patients. * bpm* beats per minute, *BID* twice daily, *DBP* diastolic blood pressure, *HR* heart rate, *LS* least-squares, *SBP* systolic blood pressure, *SE* standard error

For the cardiovascular risk markers of BP and heart rate, only BP was significantly different between treatment groups. SBP significantly decreased from baseline in the exenatide twice-daily group and increased in the glimepiride group at 36 months (*P* < 0.001) (Table 2; Fig. 2a). The between-group difference in the change from baseline was significantly in favor of exenatide twice daily at each visit from 6 to 36 months (*P* < 0.01) [7]. A significantly higher proportion of patients achieved the SBP target of <130 mmHg in the exenatide twice-daily group compared with the glimepiride group at 36 months (43.2 vs 34.7 %; *P* = 0.008). During the study treatment period, 104 of 511 (20.4 %) patients in the exenatide twice-daily group compared with 134 of 508 (26.4 %) patients in the glimepiride group received at least one new antihypertensive medication (*P* = 0.026).

DBP decreased from baseline over time in both treatment groups with the largest decrease observed in exenatide-treated patients at 36 months (Table 2; Fig. 2b). Treatment with exenatide twice daily was associated with significantly greater decreases in DBP compared with glimepiride at each visit from 6 to 18 months (*P* < 0.01) and at 36 months (*P* = 0.023). DBP was significantly lower in female versus male patients overall [LS mean (SE), −1.1 (0.27) vs 0.0 (0.25) mmHg; *P* = 0.0022]. The proportion of patients achieving the DBP goal of <80 mmHg was similar for the exenatide twice-daily and glimepiride groups at 36 months (41.2 vs 41.7 %; *P* = 0.851).

Heart rate decreased from baseline in the exenatide twice-daily group at 36 months (Table 2; Fig. 2c) but the between-group difference was not significant.

### Serum lipid changes over time in exenatide twice daily- and glimepiride-treated patients

**Fig. 3:**
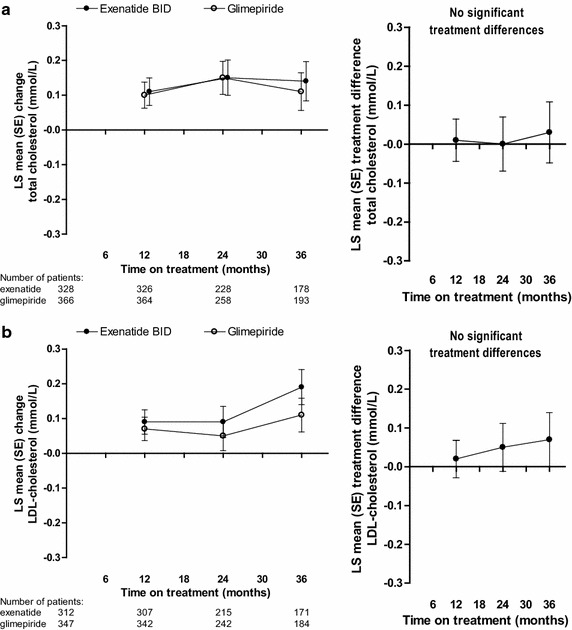
LS mean (SE) change from baseline (*left panel*) and treatment difference (*right panel*, exenatide twice daily − glimepiride) in total cholesterol (**a**) and LDL-cholesterol (**b**) in randomized patients. *BID* twice daily,* LDL* low-density lipoprotein, *LS* least-squares, *SE* standard error

Total cholesterol increased from baseline in both treatment groups at 36 months (Table 2; Fig. 3a). No significant between-group differences were observed at any time point. The change from baseline in total cholesterol was significantly greater in female versus male patients overall [LS mean (SE), 0.24 (0.04) vs 0.02 (0.03) mmol/L; *P* < 0.0001]. Serum LDL-cholesterol also increased from baseline at 36 months (Table 2; Fig. 3b). No significant between-group differences were detected at any time point. Similar to total cholesterol, the change from baseline in LDL-cholesterol was significantly greater in female versus male patients overall [LS mean (SE), 0.17 (0.03) vs 0.03 (0.03) mmol/L; *P* = 0.0015]. The proportion of patients achieving the LDL-cholesterol goal of <2.6 mmol/L was not significantly different in glimepiride-treated patients in comparison with exenatide-treated patients at 36 months (56.2 vs 51.4 %; *P* = 0.101).

**Fig. 4:**
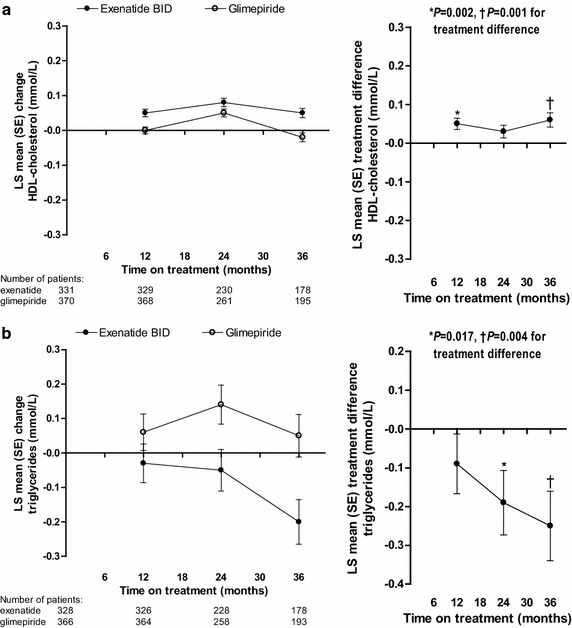
LS mean (SE) change from baseline (*left panel*) and treatment difference (*right panel*, exenatide twice daily − glimepiride) in HDL-cholesterol (**a**) and triglycerides (**b**) in randomized patients. *BID* twice daily,* HDL* high-density lipoprotein, *LS* least-squares, *SE* standard error

Serum HDL-cholesterol concentration increased slightly from baseline in exenatide-treated patients only at 36 months (Table 2; Fig. 4a). A significant between-group difference in favor of exenatide was observed at 12 (*P* = 0.002) and 36 months (*P* = 0.001). Female patients had significantly higher changes in HDL-cholesterol from baseline compared with males [LS mean (SE), 0.06 (0.01) vs 0.01 (0.01) mmol/L; *P* = 0.0006]. A significantly greater proportion of exenatide-treated patients achieved the HDL-cholesterol goal (>1.0 mmol/L for males, >1.3 mmol/L for females) than glimepiride-treated patients at 36 months (71.3 vs 64.5 %; *P* = 0.021).

Serum triglycerides decreased from baseline in exenatide-treated patients and increased in the glimepiride group at 36 months (Table 2; Fig. 4b). Significant between-group differences in favor of exenatide were observed at 24 months (*P* = 0.017) and 36 months (*P* = 0.004). The proportion of all patients achieving the triglyceride target of <1.7 mmol/L was similar in the exenatide twice-daily and glimepiride treatment groups at 36 months (54.6 vs 52.4 %; *P* = 0.514).

During follow-up, significantly fewer patients in the exenatide twice-daily group required lipid-lowering agents compared with patients in the glimepiride group (8.4 vs 12.8 %; *P* = 0.025).

### hsCRP changes over time in exenatide twice daily- and glimepiride-treated patients

Serum concentration of hsCRP decreased from baseline in both treatment groups at 36 months, with a greater decrease observed in exenatide-treated patients [mean (standard deviation), −1.7 (6.56) vs −0.2 (4.01) mg/L]. A significant between-group difference in favor of exenatide twice daily was observed at each treatment visit from 12 (*P* = 0.011) to 36 months (*P* = 0.004).

### Hypoglycemia

Documented symptomatic hypoglycemia with blood glucose <3.9 mmol/L (<70 mg/dL) was reported during 1 year of study treatment by 69 (13.5 %) and 198 (39.0 %) patients in the exenatide twice-daily and glimepiride groups, respectively (*P* < 0.0001), and during 3 years of study treatment by 98 (19.2 %) and 237 (46.7 %) patients, respectively (*P* < 0.0001). Documented asymptomatic hypoglycemia [blood glucose <3.9 mmol/L (<70 mg/dL)] in exenatide twice-daily and glimepiride treatment groups was reported by 93 (18.2 %) and 215 (42.3 %) patients, respectively, during 1 year (*P* < 0.0001), and 122 (23.9 %) and 254 (50.0 %) patients, respectively, during 3 years of study treatment (*P* < 0.0001).

## Discussion

When first-line metformin is insufficient to maintain glycemic control in patients with type 2 diabetes, practitioners are faced with an array of options for second-line add-on therapy. Either a GLP-1RA, such as exenatide, or a sulfonylurea, such as glimepiride, are viable second-line therapy options, and primary data from the long-term EUREXA study indicate that add-on exenatide twice daily provides significantly greater durability of glycemic control than add-on glimepiride [7]. Patients with type 2 diabetes are at an increased risk of cardiovascular morbidity and mortality [1]. We report that add-on exenatide twice daily was also associated with greater improvement in several cardiovascular risk markers through 36 months than add-on glimepiride. Significantly greater improvements in body weight, waist circumference, SBP, DBP, HDL-cholesterol, triglycerides, and hsCRP were observed in exenatide- compared with glimepiride-treated patients, but changes in heart rate, total cholesterol, and LDL-cholesterol were similar between treatment groups. The improvements in cardiovascular risk markers observed with exenatide twice daily mirror improvements in body weight, BP, and lipids reported from long-term treatment with exenatide once weekly for up to 5 years [10, 11].

Very few studies have compared the long-term efficacy and safety of GLP-1RAs with sulfonylureas for the improvement of glycemic control and cardiovascular markers. The Liraglutide Effect for Action in Diabetes (LEAD)-3 trial examined the effects of liraglutide 1.2 and 1.8 mg once daily compared with glimepiride 8 mg once daily for a 52-week controlled phase followed by a 52-week open-label extension [12, 13]. Similar to long-term treatment with exenatide twice daily, liraglutide 1.2 and 1.8 mg treatment for 2 years was associated with significantly improved cardiovascular risk markers including body weight and waist circumference (*P* ≤ 0.0002) [13]. Decreases in SBP and DBP and increases in heart rate were not significant in patients treated with liraglutide, while exenatide was associated with significant decreases in SBP in the present study. Changes in lipids in patients treated with liraglutide were not reported in the original LEAD-3 study or in the 52-week extension [12, 13]. However, a recent 12-month retrospective cohort study of 115 liraglutide-treated patients indicated a positive benefit on lipids, with significant reductions from baseline in total cholesterol, LDL-cholesterol, and triglycerides (*P* < 0.05), and a significant increase in HDL-cholesterol (*P* < 0.001) [14].

Differences in body weight between the two treatment groups were consistent with previous studies. The finding of weight loss with add-on exenatide twice daily in the long-term EUREXA study is supported by long-term data from two open-label extensions of clinical trials where sustained and progressive weight loss (mean change from baseline, −4.7 to −5.3 kg) up to 2–3 years’ duration were observed [15, 16]. The finding of weight gain with glimepiride treatment is also not unexpected. Data from two long-term studies observed body weight gain (mean change from baseline, 1.3 kg) with glimepiride when compared with two dipeptidyl peptidase-4 inhibitors [17, 18].

Multiple studies have examined changes in waist circumference in patients treated with add-on exenatide or glimepiride but few inform on long-term changes. Over 1 year, exenatide therapy added on to baseline metformin was associated with a significant mean reduction from baseline in waist circumference (−5 %) [19]. In a retrospective study of 179 patients treated with liraglutide or glimepiride in combination with metformin, liraglutide-treated patients had a significantly greater mean reduction in waist circumference from baseline to 18 months compared with glimepiride (−3.0 vs 0.0 cm; *P* < 0.001) [20]. One small randomized study comparing glimepiride and thiazolidinedione treatment added to metformin observed an increase in waist circumference from baseline of 1.86 cm with glimepiride [21]. In the present study we have found a difference of −4.5 cm of waist circumference at 36 months between those patients treated with exenatide versus glimepiride. This finding may be largely explained by the observed changes in weight loss; however, there is also some evidence to suggest that the positive benefits of GLP-1RA therapy on waist circumference may be related to increased serum concentrations of atrial natriuretic peptide and B-type natriuretic peptide [22].

Increased levels of hsCRP have been shown to be related to features of the metabolic syndrome [23] and incident type 2 diabetes [24], as well as with increased total atheroma volume [25] and elevated coronary heart disease mortality in patients with type 2 diabetes [26]. In the present study, both medications were associated with decreases in hsCRP through 36 months, with a greater decrease observed in the exenatide group. These findings confirm previous preliminary results from retrospective analyses [27], consistent with an association between exenatide and reduced hsCRP. It is unclear to date, however, whether this reduction in hsCRP levels is the result of the observed reversal in features of the metabolic syndrome or of a drug-specific, anti-inflammatory effect of exenatide.

Reducing BP is important in patients with type 2 diabetes to reduce risk of macrovascular and microvascular complications [28, 29]. Both SBP and DBP were reduced from baseline and maintained during 36 months of add-on exenatide therapy. The mechanism of BP reduction in patients treated with exenatide has not been clearly delineated. However, it appears that the short-term dynamics (arterial vasodilation) of BP is possibly related to the concomitant effects of reduced A1C and body weight with exenatide treatment [30, 31].

Recent studies are conflicting about the association of weight changes with improvements in BP. In the Action for Health in Diabetes (Look AHEAD) study, 1-year weight loss was found to be associated with significant improvements in SBP through 4 years [32]. When looking at individual categories of weight loss in the Practice-based Opportunities for Weight Reduction (POWER-UP) trial, patients with weight loss of 10 % or more from baseline had smaller mean improvements in SBP and DBP compared with patients with no change in weight or weight gain through 24 months [33]. At least one analysis of eight clinical trials evaluating exenatide once-weekly treatment in patients with type 2 diabetes showed greater decreases in SBP in patient quartiles with the greatest body weight loss from baseline; similar observations were also noted for reductions in LDL-cholesterol, total cholesterol, and triglycerides [34]. In a retrospective analysis evaluating liraglutide treatment over 7.5 months, significant reductions in SBP from baseline (~7 mmHg; *P* < 0.001) were observed, but the reductions were not found to be associated with body weight loss (*r* = 0.22; *P* = 0.24) [35].

BP target achievement was low overall considering that 65 to 70 % of patients were taking antihypertensive treatments at baseline. Less than half of all patients achieved the SBP (<130 mmHg) and DBP (<80 mmHg) targets [9]. One possible explanation for the low rates observed is that patients with type 2 diabetes mellitus are generally regarded as a population that is difficult to treat to guideline-recommended BP goals [36, 37]. Similar SBP goal achievement rates were observed across several clinical trials in an analysis of exenatide once weekly, although an incremental benefit was observed with exenatide compared with other oral glucose-lowering therapies [38]. Another explanation relates to the study design; there was no protocol requirement to standardize concomitant antihypertensive medications. Despite randomization, there was a disparity in the proportions of patients taking concomitant medications at baseline. It is interesting that BP reductions were greater in the exenatide twice-daily group despite less use of antihypertensive medications at baseline and a significantly lower proportion of patients receiving at least one new antihypertensive medication during the study.

Heart rate was decreased by less than 1 beat per minute in patients treated with exenatide twice daily and the lack of change in heart rate is not unexpected. In a pooled analysis of studies comparing exenatide once weekly with other glucose-lowering medications, early increases in heart rate were observed with all GLP-1RA therapies [39]. At the endpoint observation, a clinically insignificant mean increase in heart rate of 0.9 beats per minute was observed in patients treated with exenatide twice daily, compared with increases of 2.8 and 2.6 beats per minute for the longer-acting exenatide once weekly and liraglutide once daily, respectively. Inhibited neurotransmission of vagal neurons, increased sympathetic activity, and endothelium-induced vasodilation are all proposed mechanisms for heart rate increases that are observed with GLP-1RA therapy [40]. It is possible that shorter-acting exenatide twice daily has less impact on heart rate than longer-acting once-daily liraglutide or once-weekly exenatide because exenatide was present for short periods of time during the day and may not have been present when heart rate was measured [41, 42].

Total cholesterol and LDL-cholesterol increased from baseline in both treatment groups during 36 months of add-on therapy with no significant between-group differences observed, while a decrease in triglycerides and an increase in HDL-cholesterol were observed in patients treated with add-on exenatide twice daily. At baseline, the mean values for LDL- (2.5 mmol/L) and HDL-cholesterol (1.3 mmol/L) in the study population were already within recommended targets [LDL-cholesterol <2.6 mmol/L; HDL-cholesterol >1.1 (male) and >1.3 (female) mmol/L]. More than half of randomized patients were taking lipid-lowering agents at baseline. As was seen with antihypertensive medication, fewer patients on exenatide received additional lipid-lowering medication during the course of the study. At least one long-term observational follow-up study of 82 weeks’ duration showed significant improvements from baseline in mean HDL-cholesterol and triglycerides with exenatide twice-daily therapy; improvements in total cholesterol and LDL-cholesterol were not found to be significant [43]. There is a lack of long-term data on the effects of glimepiride on lipid profiles. At least one short-term study of glimepiride added on to metformin showed that glimepiride had a neutral effect on lipids overall [44].

Despite the observed differences in biomarkers, no information is yet available on any differences in cardiovascular events between these therapies. Large retrospective database analyses and meta-analyses of randomized controlled trials have suggested either decreased rates or no increased risk of cardiovascular events in patients treated with GLP-1RAs [45–47], despite evidence that GLP-1RA therapy improves lipid profiles and a wide variety of markers of endothelial and vascular health [42, 48, 49]. Prospective clinical trials such as the ongoing Exenatide Study of Cardiovascular Event Lowering (EXSCEL) (ClinicalTrials.gov identifier: NCT01144338) are required to confirm these findings. However, some data suggest that sulfonylureas have negative effects on cardiovascular risk and mortality [50–54], so a comparative cardiovascular outcomes trial between exenatide twice daily and a sulfonylurea would provide interesting results.

Limitations of this study include the open-label design, which could have led to bias due to expectations of effects on cardiovascular markers. Furthermore, the study design of EUREXA was such that patients who did not maintain glycemic control exited the study over time, thus responders may have been selected for.

## Conclusions

This secondary analysis of results from the EUREXA study showed that up to 36 months of treatment with add-on exenatide twice daily instead of glimepiride was associated with improved body weight, waist circumference, BP, HDL-cholesterol, triglycerides, and hsCRP in patients with type 2 diabetes who failed metformin monotherapy. These findings represent the longest controlled observation of the effects of exenatide twice daily on cardiovascular risk markers to date and the only long-term data (36 months) for exenatide in comparison with a sulfonylurea. These data may assist physicians in choosing a second therapy for their patients with type 2 diabetes at increased risk of cardiovascular events. In addition, the positive effects of exenatide twice daily on BP, HDL-cholesterol, and triglycerides may be associated with less antihypertensive and lipid-lowering medication use in this population. However, additional studies are needed to confirm this association and to evaluate any possible economic benefit.

## Abbreviations

BP: blood pressure
DBP: diastolic blood pressure
EUREXA: EURopean EXenAtide
GLP-1RA: glucagon-like peptide-1 receptor agonist
HDL: high-density lipoprotein
hsCRP: high-sensitivity C-reactive protein
MMRM: mixed model repeated measures
ITT: intent-to-treat
LDL: low-density lipoprotein
LS: least-squares
SBP: systolic blood pressure
SE: standard error
